# An atypical case of febrile infection-related epilepsy syndrome following acute encephalitis: impact of physiotherapy in regaining locomotor abilities in a patient with neuroregression

**DOI:** 10.11604/pamj.2020.36.101.23855

**Published:** 2020-06-17

**Authors:** Chanan Goyal, Waqar M. Naqvi, Arti Sahu

**Affiliations:** 1 Government Physiotherapy College, Raipur, India,; 2 Ravi Nair Physiotherapy College, Datta Meghe Institute of Medical Sciences, Wardha, India

**Keywords:** Post encephalitis sequelae, neurodevelopmental treatment, sensory integration

## Abstract

Encephalitis refers to inflammation of the brain parenchyma. It is potentially life-threatening with the highest incidence and severity in younger children. Febrile infection-related epilepsy syndrome (FIRES) is a condition, in which a child develops a nonspecific febrile illness that may not persist when the initial seizure activity begins. However, an electroencephalogram (EEG) shows that the child is in status epilepticus. We report the case of a five-year-old male who presented with difficulty to maintain sitting posture, and inability to stand and walk without support, following viral encephalitis at the age of one year. He had motor, visual, speech and cognitive impairment along with a seizure disorder. The physiotherapy interventions including neurodevelopmental treatment (NDT) and sensory integration (SI) helped in regaining locomotion ability in the child. The study aims to assess the impact of physiotherapy interventions on regaining locomotor ability in a child with FIRES following infective encephalitis.

## Introduction

In febrile infection-related epilepsy syndrome (FIRES), a child develops a nonspecific febrile illness, which may not persist when the seizure activity begins, that results in status epilepticus [1]. According to the estimates, 1 in 1,000,000 children develops FIRES [2]. The syndrome is supposedly caused by an inflammatory or autoimmune mechanism [2]. The prognosis of FIRES is very poor, and it remains a tough disease to treat [3]. The research is not sufficiently advanced, majorly because it is an atypical disease. An infectious condition like 'acute encephalitis' can result in FIRES. Acute encephalitis syndrome means a sudden onset of fever with seizures or alteration of consciousness or both [4]. Neurologic sequelae can result due to acute encephalitis [5]. Nearly 50 per cent of childhood survivors of infective encephalitis report incomplete long-term recovery [6]. Individualized physiotherapy treatment helps in regaining functional abilities. This study focuses on the course and outcomes of long-term rehabilitation of a child with motor deficits along with associated visual deficit, cognitive impairment, and seizure.

## Patient and observation

A 5-year-old male child, who was the firstborn of a non-consanguineous marriage, was diagnosed with a seizure disorder and neuroregression as a post encephalitis sequel. He presented at the physiotherapy department with an inability to stand and walk. He was able to hold neck, roll, and maintain sitting with support. The patient was delivered by cesarean section, cried immediately after birth, and weighed 2 kilograms. He achieved age-appropriate developmental milestones in the first year of life. On his first birthday, he performed unsupported standing, took a few steps without the support and spoke 2-3 meaningful words. At 13 months of age, he had an episode of fever. After 24 hours, he experienced convulsions and was referred to a tertiary care centre by a local practitioner. He was admitted to PICU for 15 days with a high-grade fever of 106 degrees F and refractory status epilepticus. He was afebrile after day 3 of admission, and seizures continued till day 6. At the time of discharge, he had a frequency of 10 seizure episodes per day. He had neuroregression in all domains of development. He was unable to hold neck or roll, unable to speak, and unresponsive to any visual stimulus. He was discharged with anti-epileptic medications and a home exercise program.

**Figure 1:**
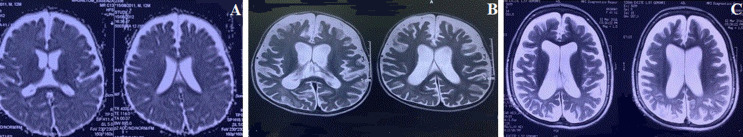
MRI (axial view) findings of the brain: (A) no significant abnormality (acute phase); (B) marked generalized cerebral atrophy and signal abnormality in the right temporoparietal regions, likely to be sequelae of encephalitis (subacute phase); (C) prominence of the ventricular system indicating cerebral atrophy (chronic phase)

On evaluation, his Gross Motor Function Classification System (GMFCS) was at level IV, and Pediatric Balance Scale (PBS) score was 5. He had generalized hypotonia, preferred W-sitting, had tightness of bilateral hamstrings and calf muscles, displayed body rocking and chewed the clothes. Child neither made eye contact nor, was there visual fixation and tracking. His light perception was equivocal. Figure 1 and Table 1 shows the Magnetic Resonance Imaging (MRI) findings of the brain and the timeline of the events, respectively.

**Table 1:** timeline of events

Time	Event	Consultation
12 July 2011	Full-term cesarean delivery, low birth weight	Obstetrician and Pediatrician
14 August 2012	Fever, medicine prescribed	Pediatrician
15 August 2012	High fever, convulsions, referred to a tertiary care centre	Pediatrician
15 August 2012	MRI of the brain showed no significant abnormality	Radiologist
15 August 2012	Admitted in PICU for refractory status epilepticus	Pediatrician
18 August 2012	Afebrile but seizures uncontrolled	Pediatrician
18 August 2012	EEG showed single generalized sharp wave discharge with slowing	Neurologist
20 August 2012	Seizures frequency reduced	Pediatrician
1 September 2012	Discharged from PICU with the diagnosis of encephalitis, prescribed medicines and home exercise program	Pediatrician and Physiotherapist
18 September 2012	EEG showed attenuated background over the right temporooccipital area and epileptiform discharges over left parieto-occipital area	Pediatric neurologist
18 September 2012	MRI of brain and MR spectroscopy revealed marked generalized cerebral atrophy and signal abnormality in right temporoparietal regions, likely to be sequelae of encephalitis	Radiologist
8 April 2013	EEG showed evolving hypsarrhythmia with multiple independent spike foci	Pediatric neurologist
7 October 2013	EEG showed multifocal epileptiform activity with secondary generalization, predominantly to left hemisphere	Neurologist
10 October 2013	Diagnosed as myoclonic epilepsy with autistic traits as a sequel of status epilepticus, prescribed medicines and home exercise program	Neurologist, Physiotherapist, Occupational therapist
12 March 2016	MRI of brain showed prominent ventricular system indicating cerebral atrophy	Radiologist
30 January 2017	GMFCS: Level IV PBS: 5 Did not fix on the light	Pediatric physiotherapist
29 April 2017	GMFCS: III PBS: 13 Visual fixation on the light inconsistently present	Pediatric physiotherapist
22 March 2018	Visual evoked potential showed delay with lower amplitude	Pediatric neurologist
19 August 2019	GMFCS: III PBS: 19 Visual fixation present but following absent	Pediatric physiotherapist
3 March 2020	GMFCS: Level II PBS: 28 Occasionally followed the light	Pediatric physiotherapist

Diagnostic assessment: GMFCS and PBS were used to measure gross motor function and balance, respectively.

Diagnostic challenges: visual and cognitive impairment posed challenges. Furthermore, the child continued to have seizures 3-4 times per day.

**Physiotherapy intervention:** the patient received regular physiotherapy based on the principles of neurodevelopmental treatment (NDT) and sensory integration (SI). The initial phase of 3 months included sustained stretching of the bilateral calf and hamstring muscles in functional positions, using unstable surfaces (equilibrium board and swiss ball) to challenge his sitting balance, using swing system to impart vestibular input, using visual stimulus to initiate a response, facilitating transitions like supine to sit and sit to stand by appropriate therapist´s hand placement, providing proprioceptive stimulus by bouncing on a trampoline with support, weight-bearing exercises, joint compressions and use of a chewy tube. As the child regained the ability to sit and stand without support, walker (rollator) was used as a walking aid. The parents were educated about the home exercise program. During the follow-up visits interspersed over 2 years, the patient was reassessed and the exercise plan was modified accordingly. The child started taking a few steps without support. In the second phase of regular physiotherapy for 6 months by a physiotherapist, the goal was to achieve unsupported walking under supervision in the view of visual impairment. Sessions included standing on equilibrium board and stability disc with just as much support as required. Walking on compliant surfaces, ramp, uneven terrain and across obstacle course was practiced. Negotiating stairs holding onto the railing with moderate assistance was included. Proprioceptive, vestibular and visual inputs were continued to be given.

**Figure 2:**
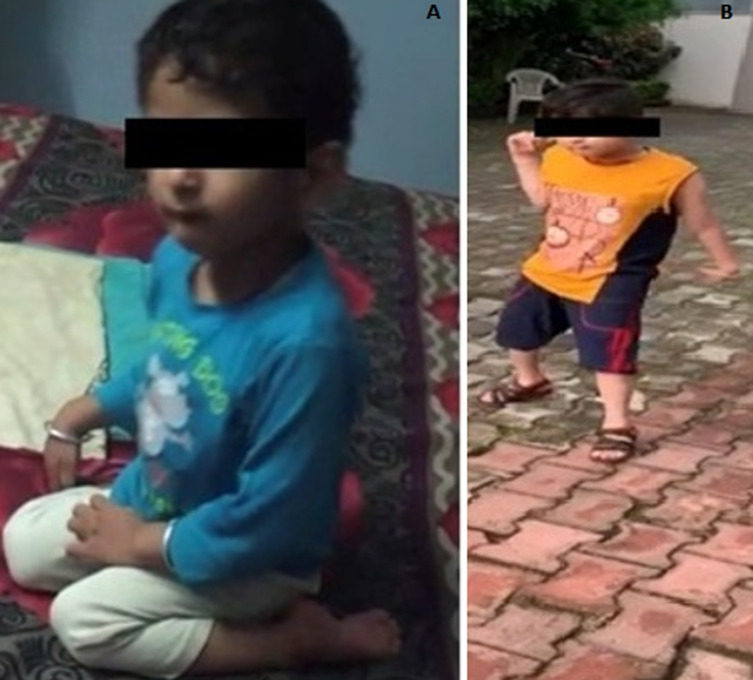
functional abilities of the child: (A) adopted W-sitting and was not able to walk at the age of 5 years; (B) independent walking at the age of 8 years

Outcomes: after the course of physiotherapy, the child was able to sit and stand without support. He was able to walk without support on uneven surfaces under standby supervision. He started responding to light. He seemed to be avoiding bumping into objects or persons by apparently improved visual ability. Body rocking and chewing on clothes were seldom observed. Improvement in functional activities led to an increase in the participation of the child in social gatherings. Figure 2 shows the functional abilities of the child at the age of 5 years and 8 years, respectively.

## Discussion

FIRES, a term often used for pediatric population though applicable for all ages, occurs due to a prior febrile infection beginning between 2 weeks and 24 hours before the onset of the refractory status epilepticus, with or without fever at the onset of status epilepticus [7]. A study conducted by Nicola Specchio and Nicola Pietrafusa in 2020, revealed that 61.2 per cent of patients with FIRES have a normal brain MRI initially, and only 18.5 per cent during the chronic phase [8]. As per the history and reports, the child fits into the criteria for FIRES, a rare diagnosis. The achievement of gross motor milestones can be attributed to the active participation of the child in learning the skills through facilitation by physiotherapist based on principles of NDT. SI approach must have contributed to improving his balance [9]. Vestibular stimulation helps in visual system facilitation through vestibular-visual interaction [10]. Proprioceptive input must have contributed to reducing the chewing on clothes and body rocking along with vestibular input. Similar delayed regaining of walking abilities in patients with post encephalitis sequelae has been reported in the previous study [11].

## Conclusion

The study concluded that physiotherapy interventions based on principles of NDT and SI, markedly improved child´s gross motor skills and balance as observed by GMFCS and PBS respectively. Also, improvement in the visual ability was observed that can be associated with vestibular-visual interaction. This study opens up new avenues for further studies to understand the role of physiotherapy in regaining sensory-motor functions in patients with FIRES.
